# Current status of type 1 (IgG4-related) autoimmune pancreatitis

**DOI:** 10.1007/s00535-022-01891-7

**Published:** 2022-08-02

**Authors:** Kazushige Uchida, Kazuichi Okazaki

**Affiliations:** 1 Department of Gastroenterology and Hepatology, Kochi Medical School, Kochi University, Okocho-Kohasu, Nankoku, Kochi 783-8505 Japan; 2 Kansai Medical University Kouri Hospital, 8-45 Kourihondori, Neyagawa, Osaka 572-8551 Japan

**Keywords:** IgG4, Autoimmune pancreatitis, Regulatory *T* cells, M2 macrophage, Basophil

## Abstract

In 1995, Yoshida et al. proposed first the concept of “autoimmune pancreatitis” (AIP). Since then, AIP has been accepted as a new pancreatic inflammatory disease and is now divided two subtypes. Type 1 AIP affected immunoglobulin G4 (IgG4) and implicates the pancreatic manifestation of IgG4-related disease, while type 2 is characterized by neutrophil infiltration and granulocytic epithelial lesions (GEL). Recent research has clarified the clinical and pathophysiological aspects of type 1 AIP, which is more than type 2 among the Japanese population. However, many details remain unclear about the pathogenesis and progression of this disease. In this review, we discuss the current knowledge and recent advances relating to type 1 AIP.

## History of autoimmune pancreatitis and immunoglobulin G4-related disease: the pre and post recognition of IgG4

In 1961, the first published case of chronic pancreatitis with hypergammaglobulinemia was reported [1]. This report is thought to be the first relating to autoimmune pancreatitis (AIP).

Thirty years later, in 1991, Kawaguchi et al. described and proposed lymphoplasmacytic sclerosing pancreatitis (LPSP) from a resected mass in the context of chronic pancreatitis, which is clinically difficult to distinguish from pancreatic cancer. Histologically, LPSP is characterized by lymphoplasmacytic infiltration, storiform fibrosis, and obliterative phlebitis. These features represent the pathological basis of the disease that is referred to as AIP [2].

In 1995, Yoshida et al. proposed the concept of AIP [3]. Following their proposal, in 2001, Hamano et al. described that Japanese patients with AIP have increased serum levels of immunoglobulin G4 (IgG4), considered as the first report of IgG4-related disease (RD) [4]. Several Japanese investigators have since described the clinical features of AIP [5–8]. In 2003, Kamisawa et al. proposed that AIP is a systemic disorder that was named as “IgG4-related sclerosing disease” [9]. This propose was on the basis of the histological features of extrapancreatic lesions in other organs—similar to that seen in LPSP—and the presence of abundant infiltration of IgG4-positive plasma cells [9].

Another historical description of IgG4-RD was the report of Mikulicz’s disease, which was described in 1892 by Johan Freisherr von Mikulicz-Radecki as symmetrical swelling of the lachrymal, parotid and submandibular glands with abundant infiltration of mononuclear cells [10]. This report was published about 70 years ago from Sarle’s pancreatic case report [1]. However, Mikulicz’s disease had since been classified as a subtype of Sjogren’s syndrome [11]. Yamamoto et al. suggested Mikulicz’s disease to be an IgG4-related plasmacytic disease [12], and the authors proposed the term “IgG4-related plasmacytic syndrome (SIPS)” [13]. In 2008, Masaki et al. also suggested the term “IgG4-multiorgan lymphoproliferative syndrome (IgG4-MOLPS)” based on the lymphoproliferative disorder [14]. Thus, three separated groups of Japanese investigators have proposed different names for the systemic disease that involves IgG4 as the differ for each. In response to these proposals, Japanese research groups (Research Program for Intractable Disease of the Japan Ministry of Health, Labor, and Welfare of Japan) unified these concepts to include IgG4-related AIP, IgG4-related sclerosing cholangitis, Mikulicz’s disease, and other IgG4-related conditions and proposed the term as IgG4-RD [15]. In 2011, the first international symposium on IgG4-RD was held in Boston and accepted this concept [16].

During the time that IgG4-related autoimmune pancreatitis (LPSP) was being increasingly reported on in Japan and becoming more recognized worldwide, western countries began reporting a different type of AIP with histological characteristics quite different to those of LPSP. In 2003, Notohara et al. described idiopathic duct-centric pancreatitis (IDCP), characterized by neutrophilic infiltration within the lumen and epithelium of the interlobular ducts [17]. Another name is AIP with granulocyte epithelial lesions (AIP with GEL) [18]. In 2011, the International Consensus Diagnostic Criteria for Autoimmune Pancreatitis (ICDC) published that AIP could be classified into type 1 AIP (LPSP) or Type 2 AIP (IDCP) [19]. Based on the ICDC, the clinical diagnostic criteria of AIP 2011 were submitted for general Gastroenterologist by the Japan Pancreas Society (JPS), the Ministry of Health and Welfare of Japan Investigation Research Team for Intractable Pancreatic Disease [20]. Recently, it was revised as proposed in the Clinical Diagnostic Criteria of Autoimmune Pancreatitis 2011, which has since been revised as the Japanese Clinical Diagnostic Criteria for Autoimmune Pancreatitis, 2018 (Proposal)-Revision of Japanese Clinical Diagnostic Criteria for Autoimmune Pancreatitis, 2011 [21].

## Epidemiology

The prevalence of type 1 AIP in Japan is 10.1/100,000 with an incidence of 3.1 per 100,000 according to a 2016 nationwide survey in Japan, which also revealed the mean age of patients to be 68.1 years [22]. The total number and number of newly diagnosed patients with type 1 AIP had increased by 2.3-fold and 2.2-fold, respectively, compared with a 2011 nationwide survey [23]. Type 2 AIP is uncommon in Asian countries, including Japan; a multinational analysis involving 23 institutions from 10 different countries reported that type 2 AIP records for 3.7 and 12.9% of all AIP cases in Asian and European countries/USA, respectively [24]. An Italian multicenter study showed that the mean age of patients was 62.5 and 48 years for type 1 and type 2 AIP, respectively, and the proportion of cases that were male was 66.9 and 54.2%, respectively [25]. However, while there appears to be considerable information on the incidence of AIP, the exact prevalence of type 1 and type 2 AIP worldwide remains unknown.

## Diagnosis of type 1 autoimmune pancreatitis

### Symptoms

It has been provided that 62.8% of patients with type 1 AIP are symptomatic in the 2016 Japanese nationwide survey; among these, 48.6% showed jaundice, 25.6% experienced abdominal pain, and 12% reported other symptoms involving extrapancreatic lesions [22]. These symptoms were mainly related to sialadenitis and dacryoadenitis. Acute pancreatitis was only identified in 0.9% of symptomatic cases.

Among the asymptomatic cases of type 1 AIP, 57.8% exhibited abnormal imaging findings, 23.4% abnormal laboratory data, and 18.8% had new-onset or exacerbated diabetes mellitus [22].

### Serology

The validity of serum IgG4 concentration was confirmed, with a cutoff value of 135 mg/dL [15, 20, 21]. The 2016 Japanese survey revealed 84.5% of patients with type1 AIP who have shown high serum IgG4 [22]. However, elevated serum levels of IgG4 are detected in other diseases (e.g., bronchial asthma, atopic dermatitis, pemphigus, and multicentric Castleman’s disease), and has been reported to be a feature of about 10% of cases of malignancy (e.g., pancreatic cancer and cholangiocarcinoma) [15]. In the 2016 Japanese nationwide survey, other serological markers elevated IgG are detected in 58.5% patients, anti-nuclear antibody in 32.6%, and rheumatoid factor in 18.3% [22]. However, a specific marker of type 1 AIP still has not been found.

### Radiological imaging

Diffuse pancreatic swelling and irregular narrowing of the main pancreatic duct (MPD) are characteristics of AIP. On dynamic computed tomography (CT) and contrast-enhanced magnetic resonance imaging (MRI), contrasting patterns are characterized by a capsule-like rim and delayed enhancement. These findings are associated with fibrosis of the pancreas. Other findings on MRI are often seen as low signal intensity on fat-suppressed T1-weighted imaging and speckled/dotted enhancement. However, low signal intensity on fat-suppressed T1-weighted imaging is not specific characteristic of AIP. Endoscopic retrograde cholangiopancreatography (ERCP) typically shows a narrowing of the MPD over more than one-third of its length. In the segmental/focal type, side branches arising from the stricture area and narrowing ducts without upstream dilatation are important findings that distinguish pancreatic cancer. With magnetic resonance cholangio-pancreatgraphy (MRCP), the narrowing of the MPD is not clearly visible, and the MPD is shown as multiple intermittent (skipped) absence [26].

With fluorodeoxyglucose-positron emission tomography (FDG-PET), the accumulation of FDG is detected in not only the pancreas and but also in extrapancreatic lesions. This accumulation reduces or disappears after steroid therapy. Nevertheless, FDG-PET is not covered by Japanese medical insurance [26].

### Diagnostic criteria for autoimmune pancreatitis in Japan

In 2002, the JPS published first the diagnostic criteria for AIP in the world [27]. Serum levels of IgG4 were not included in this diagnostic criteria, because it was considered that elevated serum levels of IgG4 for diagnostic item was premature.

As clinical features of AIP became more understand, two problems arose; first, while the histological features of LPSP and IDCP were clearly different, it was impossible to distinguish between the two with imaging findings alone. Secondly, diagnostic methods differ across the world; as an example, pancreatograms obtained by ERCP had been historically considered to be important in the diagnosis of AIP in Japan and Korea [28]. In contrast, ERCP is not generally employed for the diagnosis of pancreato-biliary disease in western countries. To address these issues, the International Consensus Diagnostic Criteria for AIP (ICDC) was proposed in 2011 [19]. Firstly, LPSP and IDCP were termed type 1 AIP and type 2 AIP, respectively (Table 1). The ICDC assessed type 1 AIP using a combinatorial approach which considered: (i) imaging characteristics of (a) parenchymal imaging (pancreatic enlargement) and (b) ductal imaging (ERP); (ii) serology (serum levels of IgG4); (iii) other organ involvement; (iv) histology of the pancreas; and (v) response to steroid therapy. Additionally, these diagnostic elements are classified in two levels, such as level 1 and level 2 (Table 2). The diagnosis of type 2 AIP (IDCP) considers only four of the above features, with serology (IgG4) excluded. Therefore, there are various findings that must be present for a diagnosis of ICDC to be made, which complicate the diagnostic criteria for general gastroenterologists.

**Table 1:** Characteristics of type 1 and type 2 autoimmune pancreatitis

	Type1	Type2
Prevalence	Asian > caucasian	Caucasian > asian
Age	Older	Younger
Gender	Male > > female	Male = female
Clinical findings	Jaundice (painless)	Abdominal pain
		Jaundice
Swelling of the pancreas	Common	Common
Serum levels of IgG4	Elevated	Normal
Histology	LPSP	IDCP
OOI	Cholangitis	Ulcerative colitis
	Sialadenitis	
	Kidney lesion	
	Retroperitoneal fibrosis	
	Others (IgG4-RD)	
Responsiveness to steroid	Good	Good
Relapse	Often	Rare

*IDCP* idiopathic duct-centric pancreatitis, *Ig* immunoglobulin, *LPSP* lymphoplasmacytic screlosing pancreatitis, *OOI* other organ involvement, *RD* related disease

**Table 2:** Comparison of International Consensus Diagnostic Criteria for Autoimmune Pancreatitis (ICDC) and Japanese Clinical Diagnostic Criteria for Autoimmune Pancreatitis, 2018 (JPS 2018)

	ICDC		JPS 2018
	Level 1	Level2	
Imaging of CT or MRI	Diffuse enlargement with delayed enhancement	Segmental/focal enlargement with delayed enhancement	Diffuse enlargement, segmental/focal enlargement
Pancreatogram	Long or multiple strictures without marked upstream dilatation by ERP	Segmental/focal narrowing without marked upstream dilatation by ERP	Irregular narrowing of MPD by ERP or MRCP
Serum levels of IgG4	> 2X	1-2X	≧ 135 mg/dl
Histology	1. Marked lymphoplasmacytic infiltration with fibrosis and without granulocytic infiltration 2. Storiform fibrosis 3. Obliterative phlebitis 4. Abundant (> 10 cells/HPF) IgG4-positive cells	1. Marked lymphoplasmacytic infiltration with fibrosis and without granulocytic infiltration 2. Abundant (> 10 cells/HPF) IgG4-positive cells	1. Prominent infiltration of lymphocytes and plasma cells along with fibrosis 2. More than 10 IgG4–positive plasma cells per high–power microscopic field 3. Storiform fibrosis 4. Obliterative phlebitis < definite: three or more of 1 ~ 4 are observed >
Extrapancreatic lesions	Sclerosing cholangitis retroperitoneal fibrosis	Symmetrical enlarged salivary/lacrimal glands renal involvement	Sclerosing cholangitis sclerosing dacryoadenitis/sialoadenitis retroperitoneal fibrosis kidney lesion
Response of steroid	Rapidly (< 2 weeks)		No neoplastic cells detectable by EUS-FNA is necessary

*CT* computed tomography, *MRI* magnetic resonance imaging, *ERP* endoscopic retrograde pancreatography, MRCP magnetic resonance cholangio-pancreatgraphy, *MPD* main pancreatic duct, *EUS-FNA* endoscopic ultarasound-guided fine-needle aspiration

Taking ICDC, the JPS and the Ministry of Health and Welfare Investigation Research Team for Intractable Pancreatic Disease proposed clinical diagnostic criteria of AIP 2011 (JPS 2011). JPS 2011 is on the basis of the ICDC and a simplified checklist of diagnostic items for type 1 AIP because the majority of AIP is type 1 in Japan. Japanese diagnostic criteria from the first JPS2002 were aimed at general gastroenterologists to prevent misdiagnosis of pancreatic cancer as AIP. JPS2018 has been proposed (Table 3) after the revision of JPS2011 [21]. The concept of JPS2018 hand over from JPS2011. The main checklists of the JPS2018 are as follows: (i) in diffuse type 1 AIP, the pancreatogram is not essential; but in segmental/focal-type type 1 AIP, ERCP or MRCP is essential; (ii) serum levels of IgG4 (≧ 135 mg/dl); (iii) histology; (iv) extrapancreatic lesions (sclerosing cholangitis, sclerosing dacryoadenitis/sialadenitis, retroperitoneal fibrosis, kidney disease) indicated by clinical or histological findings; and (v) effectiveness of steroid therapy with the avoidance of pancreatic cancer using endoscopic ultrasound-guided fine-needle aspiration (EUS-FNA) [21].

**Table 3:** Japanese Clinical Diagnostic Criteria for Autoimmune Pancreatitis, 2018 [21]

Diagnostic criteria
A. Diagnostic items
I. Enlargement of the pancreas
a. Diffuse enlargement
b. Segmental/focal enlargement
II. Imaging findings showing irregular narrowing of the main pancreatic duct
a. ERP (endoscopic retrograde pancreatography)
b. MRCP (magnetic resonance cholangiopancreatography)
III. Serological findings
Elevated serum IgG4 (≧ 135 mg/dl)
IV. Pathological findings among 1 ~ 5 listed below
a. Three or more of 1 ~ 4 are observed
b. Two of 1 ~ 4 are observed
c. 5 is observed
1. Prominent infiltration of lymphocytes and plasma cells along with fibrosis
2. More than 10 IgG4-positive plasma cells per high-power microscopic field
3. Storiform fibrosis
4. Obliterative phlebitis
5. No neoplastic cells detectable by EUS-FNA (endoscopic ultrasound-guided fine-needle aspiration biopsy)
V. Extrapancreatic lesions including sclerosing cholangitis, sclerosing dacryoadenitis/sialoadenitis, retroperitoneal fibrosis, and kidney lesion
a. Clinical lesions
Extrapancreatic sclerosing cholangitis, sclerosing dacryoadenitis/sialoadenitis (Mikulicz disease), retroperitoneal fibrosis, or kidney lesion detectable by clinical and imaging findings
b. Pathological lesions
Pathological examination showing characteristic features of sclerosing cholangitis, sclerosing dacryoadenitis/sialoadenitis, retroperitoneal fibrosis, or kidney lesion
VI. Effectiveness of steroid therapy
A specialized facility may include in its diagnosis the effectiveness of steroid therapy once pancreatic and bile-duct cancers have been ruled out. When it is difficult to differentiate malignant conditions, cytological examination using EUS–FNA (IVc) is desirable. Facile therapeutic diagnosis by steroid responsiveness alone should be avoided unless the possibility of malignant tumor has been ruled out by pathological diagnosis. Accordingly, VI includes IVc

B. Diagnosis
I. Definite diagnosis
1. Diffuse type
Ia + < III/IVb/V (a/b) >
2. Segmental/focal type
Ib + IIa + two or more of < III/IVb/V (a/b)
or
Ib + IIa + < III/IVb/V (a/b) > + VI
or
Ib + IIb + < III/V (a/b) > + IVb + VI
3 Definite diagnosis by histopathological study IVa
II. Probable diagnosis
Segmental/focal type: Ib + IIa + < III/IVb/V (a/b) >
or
Ib + IIb + < III/V (a/b) > + IVc
or
Ib + < III/IVb/V (a/b) > + VI
III. Possible diagnosis*
Diffuse type: Ia + II (a/b) + VI
Segmental/focal type: Ib + II (a/b) + VI

*Possible diagnosis: a case may possibly be type 2, although this is extremely rare in Japan. For section B, “ + ” indicates “and” and “/” indicates “or”

### Histopathological diagnosis

Histopathological characteristics of type 1 AIP are mentioned previously. The use of EUS-FNA is increasing and may become common endoscopic procedures. Indeed, the 2016 Japanese nationwide survey revealed that tissue sampling using EUS-FNA/biopsy (EUS-FNA/B) was carried out in 85.5% of cases of AIP [22]. Especially, EUS-FNA/B is useful in case of focal type of AIP or within normal limit of serum levels of IgG4. The technique is useful for exclusion of pancreatic cancer; however, it is sometimes difficult to diagnose AIP due to difficulties associated with the small samples that are involved. Specifically, several investigators have already reported the presence of IgG4-positive cells in pancreatic ductal adenocarcinoma [29, 30], and the infiltration of neutrophils into the pancreas with type 1 AIP which is characteristic of type 2 AIP [31]. The comprehensive diagnostic criteria of IgG4-RD include infiltration of IgG4-positive cells (defined as a ratio of IgG4-positive plasma cells/IgG-positive cells of > 40%, and > 10 IgG4-positive plasma cells per high-power field (hpf)) [32]. Fukui et al. investigated the presence of IgG4-positive cells in pancreatic ductal adenocarcinoma in relation to the comprehensive diagnostic criteria of IgG4-RD [33]. They found the ratio of IgG4/IgG to be > 40% in 43, 29, and 14% of primary cancer lesions, peritumoral pancreatitis lesions and obstructive pancreatitis lesions, respectively. The incidence of > 10 IgG4-posotive cells per hpf was 5 and 10% among samples from primary cancer lesions and obstructive pancreatitis lesions, respectively. In type 1 AIP, 89% of cases satisfied an IgG4/IgG ratio of > 40% and > 10 IgG4-positive cells per hpf. However, in 5% of patients with pancreatic cancer, examining primary cancer lesions and obstructive pancreatitis lesions also fulfilled these two criteria.

Neutrophil infiltration is greatest feature of type 2 AIP. Although type 2 AIP is thought to be rare in Japan, there have been reports of the cases of type 2 AIP, which can be diagnosed using EUS-FNA [34, 35]. In contrast, it has been reported that there is no statistical difference in neutrophil infiltration around the intralobular pancreatic ducts drawing a comparison between type 1 and 2 AIP. Moreover, GELs have been also observed in the intralobular pancreatic ducts in type 1 AIP case [31]. Taken together, these findings emphasize the importance of evaluating the infiltration of IgG4-positive plasma cells or neutrophils using a small biopsy obtained by EUS-FNA/B for attempting to diagnose AIP.

Another reportedly useful diagnostic tool is IgG4 immunostaining of the duodenal papillary obtained from biopsy specimens. In patients with type 1 AIP, swelling of the main duodenal papilla was first reported in 2002 by Ueno et al. [36]. Yoon SB et al. reported systematic reviews and meta-analysis of the availability of immunohistochemical staining for IgG4 in the diagnosis of AIP. IgG4 staining from the biopsy specimens of main duodenal papilla has a high specificity of the diagnosis of AIP likewise pancreatic and biliary tissues [37].

### Treatment for type 1 autoimmune pancreatitis

Steroid therapy has been established as the first-line treatment for type 1 AIP. The Japanese consensus guideline and international consensus for treatment both recommend that all active and symptomatic patients with untreated AIP receive steroid therapy [26, 38]. A rapid response to glucocorticoids is a primary feature of type 1 AIP, and the 2016 Japanese nationwide survey has reported that 98.6% of cases with type 1 AIP respond to steroid therapy [22]. An inadequate response to steroid therapy might indicate that the diagnosis of AIP should be reconsidered, and clinicians should be particularly cautious of misdiagnosis of pancreatic cancer. The Japanese consensus guidelines recommend an initial dose of oral prednisolone of 0.6 mg/kg/day, administered for 2–4 weeks, for the induction of remission [26]. However, the most generally administrated initial daily dose of prednisolone has been reported to be 30 mg (in 63.9% of cases) in the 2016 Japanese nationwide survey [22]. Imaging evaluations should be repeated after 2 weeks of therapy and the dose then tapered by 5 mg every 1–2 weeks until a maintenance dose (5.0–7.5 mg/day) is reached. A total therapy period of 3 years is recommended to prevent relapses in Japanese consensus guideline [26]. In western nations, steroid treatment is commonly limited to induction remission without maintenance therapy because of ongoing concerns about the risks of adverse events, for instance, infections, diabetes mellitus, osteoporosis, and cataracts due to steroid administration [39]. However, a randomized, controlled, multicenter study in Japanese multicenter reported that the relapse rate of the case with maintenance steroid therapy (total 3 years) to be significantly less (only occurring in 23.3% of cases) compared with after discontinuation of maintenance therapy (57.9% of cases) [40].

In spite of the high initial remission rates, it has been suggested that 15–60% of patients will experience relapse after cessation of steroid therapy or during weaning [41–43]. In the 2016 Japanese nationwide survey, 23.4% cases relapsed [22]. Detlefsen S et al. reported the relapse rate in operated patients with AIP in a European multicenter study. The total number of patients was 114. There were 63 patients with type 1 AIP and 51 with type 2 AIP that underwent operations. The relapse rates of type 1 AIP and type 2 AIP were 41.2 and 15.4%, respectively [44]. In type 1 AIP, the relapse rate was high, even among operated patients. Several factors have been reported as being predictors of relapse, including discontinuation of steroid treatment within a short period, high serum levels of IgG4 at the time of diagnosis, persistent high serum levels of IgG4 following steroid treatment, diffuse pancreatic enlargement, bile-duct lesions, and multiple extrapancreatic lesions [26]. However, it can be still difficult to predict relapse.

Rituximab is a monoclonal anti-CD20 antibody which has been reported to be a successful approach for the treatment of IgG4-RD, including type 1 AIP [45, 46]. In a clinical trial, rituximab showed efficacy for the treatment of IgG4-RD even without concomitant steroid therapy [47]. These findings suggest that B cells might be involved in the pathogenesis of IgG4-RD. However, Rituximab is not yet permitted for use in the treatment of IgG4-RD in Japan due to Japanese medical insurance reasons.

Readministration of prednisolone or administration of steroid therapy at a higher dose is the recommended course for relapse [38]. In western country, concomitant administration of immunomodulatory drugs such as azathioprine (AZA), methotrexate, and mycophenolate mofetil has been shown to be beneficial in the case of relapse or steroid resistance in patients with type 1 AIP [42, 48, 49]. A recent systematic review and meta-analysis reported the clinical efficacy of AZA as maintenance therapy [50]; however, the only immunomodulatory drugs cannot show a sufficient level of effectiveness as single agents for the induction of remission. Immunomodulatory drugs are also not approved by Japanese medical insurance for use for IgG4-RD. Thus, the development of second-line therapy for Japanese patients with type 1 AIP who suffer relapse remains essential.

### Prognosis of type 1 autoimmune pancreatitis

Steroid therapy can improve pancreatic exocrine function in type 1 AIP by suppressing inflammation and fibrosis, and regeneration by correcting aberrant localization of the cystic fibrosis transmembrane conductance regulator (CFTR) in the pancreatic duct cells, and stimulating the regeneration of acinar cells [51]. Endocrine function is also improved by steroid therapy [26] and, as a result, the prognosis of type 1 AIP is usually good in the short term.

However, the long-term prognosis is not clear due to the multiple unknown elements in particular, relapse, pancreatic exocrine/endocrine dysfunction, and accompanying malignancies as an example, pancreatic cancer. The rate of patients with type 1 AIP to develop chronic pancreatitis are reported from 7 to 40% [24, 52–55], with pancreatic head swelling and non-narrowing MPD reported to be risk factors [55, 56].

Understanding the relationship of type 1 AIP with pancreatic cancer is important because chronic pancreatitis has been reported to be a risk factors for pancreatic cancer [57]. Ikeura et al. reported that patients with type 1 AIP have a potent of higher risk for pancreatic cancer, equivalent to patients with usual chronic pancreatitis [58]. Among patients with type 1 AIP, it was also found that the K-*ras* mutation occurred not only in the pancreas but also in the bile duct and the gallbladder, frequently and significantly [59]. On the other hand, Shiokawa et al. reported the risk of developing malignancies to be highest during the first year after or before diagnosis of type 1 AIP [60]. They speculated that type 1 AIP may be a manifestation of paraneoplastic syndrome, but the definitive risk of malignancy remains unclear. Recently, a Japanese nationwide epidemiological survey of IgG4-RD with malignancy reported the frequencies of type1 AIP, IgG4-related sialadenitis, IgG4-related eye disease, IgG4-related kidney disease, and IgG4-related retroperitoneal fibrosis to be 44.7, 20.8, 14.0, 5.16, and 5.12%, respectively [61]. The overall prevalence of malignancy in IgG4-RD was estimated to be 10,900 per 100,000 cases, which was significantly higher compared with malignancy in the general population in Japan according to the National Cancer Center Japan (which reported 1,834 per 100,000 people) (*p* < 0.001). When we examine type 1 AIP patients, we always have to pay attention to malignancy include pancreatic cancer during and after steroid therapy.

## Pathophysiology of type 1 autoimmune pancreatitis

### Immunoglobulin G4

Recent researches have indicated the roles of multipathogenic factors (e.g., genetic factors, innate immunity and acquired immunity, etc.) in the development or relapse of type 1 AIP. Nevertheless, the pathogenetic mechanism of type 1 AIP still remains unclear. Among the four subclasses of IgG, IgG4 accounts for the smallest proportion (5%) of the total serum IgG [62]. The classes and subclasses of IgG are distinguished by the amino acid sequence of their heavy-chain constant domains. The differences in the CH2 domain of IgG1 and IgG4 are responsible for the difference in binding affinity to C1q and Fcγ receptors, which is negligible in the case of IgG4 [63, 64]. Most unique characteristic of IgG4 is its capability to construct half-antibodies through “Fab-arm exchange,” which involves the substitution of a heavy chain with an attached light chain [65]. Amino acid variation at the hinge region of IgG4 results in asymmetric antibodies consisting of half-antibody fragments which can then recognize two different antigens. Asymmetric IgG4 is unable to crosslink antigens and form immune complexes. These differences in structure and function might give rise to the anti-inflammatory functions of IgG4. However, IgG4 autoantibodies have been proven to be pathogenic in MuSK myasthenia gravis, chronic inflammatory demyelinating polyneuropathy, acquired thrombotic thrombocytopenic purpura, and pemphigus [66]. Shiokawa et al. reported a negative data of pathogenic activity of IgG4 in the context of type 1 AIP and IgG4-related kidney disease, and found that injection of IgG1 or IgG4 from patients with type 1 AIP could induce pancreatic injury in neonatal male Balb/c mice, with the former causing more serious pancreatic injury [67]. However, simultaneous injection of IgG4 was found to inhibit the pathogenic activity of IgG1 and the severity of pancreatic injury, highlighting the anti-inflammatory role of IgG4 in IgG4-RD including type 1 AIP.

### Genetical disorders

It has been clarified that the several genetic susceptibility to type 1 AIP as follows, the class II antigen haplotype of the major histocompatibility complex (HLA-DRB1*0405-DQB1*0401) [68] and, polymorphisms of Fc-receptor-like 3 genes [69] and cytotoxic-T lymphocyte antigen-4 gene (CTLA-4) [70, 71]. Toll-like receptor (TLR) 4 polymorphisms have already been reported in asthma and atopic dermatitis [72, 73]. Polymorphisms in the TLR4 gene were not found to be significantly associated with either susceptibility or relapse in type 1 AIP compared with healthy controls [74]. In 2009, the Japanese Research Group has characterized two susceptibility loci, HLA-DRB1 and FCGR2B, via a genome-wide association study of 857 Japanese patients with IgG4-RD [75] (Table 4).

**Table 4:** Key players in type 1 autoimmune pancreatitis (include IgG4-related disease)

Factors	Items	Increase and decrease	References
*T* cells	nTregs	↓	[83]
	eTregs	↑	[83]
	Tfh (Tfh2)	↑	[90–93]
	CD4/CD8 CTLs	↑	[94]
*B* cells	CD19 + CD27 + CD20-CD38 hi plasmablasts	↑	[95]
	CD19 + CD24 + CD38 high Bregs	↑	[97]
	CD19 + CD24 high CD27 + Bregs	↓	[97]
Innate immune cells	basophil	Present	[110]
	TLR-7-positive M2-macrophages	↑	[112]
	Neutrophil	Present	[31, 114]
Cytokines	Th2 cytokines		[76–79]
	BAFF		[108, 109]
	IL-13		[109]
	IL-35		[86]
Recent candidate of autoantigen	Prohibitin		[99]
	Annexin A11		[100]
	Laminin 511-E8		[101]
	Galectin-3		[102]
	IL-1 receptor antagonist		[103]
Gentic	HLA-DRB1		[75]
	FCGR2B		[75]
	HLA-DRB1*0405-DQB1*0401		[68]
	*FCRL3*		[69]
	*CTLA4*		[70, 71]
	*Mst1* (eTregs)		[87, 88]

*nTregs* naïve regulatory *T* cells, *eTregs* effector regulatory *T* cells, *Tfh* follicular helper *T* cells. *Bregs* regulatory *B* cells, *TLR* toll-like receptor, *FCRL3* Fc receptor-like 3, *CTLA4* cytotoxic T-lymphocyte antigen-4

## Acquired immune system

### *T* cells

*T* cells are generally divided three major types: helper *T* cells (Th), regulatory *T* cells (Tregs), and cytotoxic *T* cells (Tc). The Th1/Th2 immune balance traditionally has a crucial influence on acquired immunity. From the view of the cytokine profile of IgG4-RD includes Th2 cytokines (IL-4, IL-5, and IL-13) and regulatory cytokines (IL-10 and tumor growth actor [TGF]-β), the Th2 immune response play a key role in the pathophysiology of type1 AIP including IgG4-RD [76–79].

Tregs are classified into two major groups on the basis of their developmental origin: thymus-derived Tregs (tTregs), which are resting cells and encounter antigens in the periphery, and activated effector Tregs (eTregs) which arise from the proliferation and differentiation of tTregs. Peripherally derived Tregs (pTregs) are a minor subgroup, originating from conventional peripheral CD4^+^ *T* cells which express Foxp3, and have suppressive activity. Activated eTregs include Tregs derived from tTregs and pTregs, and while it is not possible to distinguish the origin of these Tregs [80], the expression of CD45 RA can be used to distinguish naïve (resting) Tregs from eTregs [81, 82]. In type 1 AIP, the levels of circulatory naïve (CD4^+^CD25^+^CD45RA^+^) Tregs are significantly reduced, although CD4^+^CD25^high^ eTregs are significantly enhanced in the peripheral blood. These increased eTregs is positively correlated with serum levels of IgG4 [83]. Furthermore, increased numbers of inducible costimulator (ICOS)-positive Tregs may affect IgG4 production via IL-10 in the context of type 1 AIP, while ICOS-negative Tregs may control fibrosis via TGF-β [84].

In 2007, Collison et al. reported IL-35 to be a potent immunosuppressor and anti-inflammatory cytokine produced by Tregs [85]. This cytokine is a part of the IL-12 family and stimulates the development of IL-35-producing Tregs and the expansion of regulatory B cells (Bregs). The two subunits of IL-35 are named Epstein-Barr-virus-induced 3 (EBi3) and IL-12A p35. Ito et al. reported that serum levels of IL-35 is increased and its subunits (EBi3 and IL-12A p35) are expressed in the pancreatic tissue in patients with type 1 AIP. In type 1 AIP, IL-35 might play an anti-inflammatory role and lead to the differentiation of Tregs and Bregs [86]. The production of IgG4 may be related to overexpression of anti-inflammatory cytokines such as IL-10 and IL-35, suggesting that IgG4 does not act as a pathogenic factor, or is it an anti-inflammatory factor in type 1 AIP.

These reports lead us to question why the increase in eTregs does not suppress disease activity in the case of type 1 AIP. It may be that the cell–cell contact that Tregs require to carry out their immuno-suppressing activities is inhibited. Integrin (LFA-1), which is regulated by the *Mst1* gene, is a key molecule in Rap1- and RapL-mediated cell–cell contact, and knockout of this gene has been shown to cause sialadenitis and pancreatitis reflective of IgG4-RD in mice [87]. Fukuhara et al. investigated the role of *Mst1* in type 1 AIP and found that patients exhibit decreased expression of MST1 in Tregs compared with healthy controls [88]. The frequency of methylated CpG sites in *MST1* was significantly increased among patients with type 1 AIP and extrapancreatic lesions, which was found to be correlated with the number of affected organs. These results reveal a putative role of *MST1* in the pathological mechanism underlying the progression of IgG4-RD.

It is well known that CD4 T cells play important roles in the formation of germinal centers and the differentiation of memory *B* cells and plasmacytes into secondary lymphoid tissue. Recently, CXCR5 + CD4 T cells—which are located in the germinal center—have been found to be involved in T-cell-dependent antibody production, and have thus been named follicular helper T cells (Tfh) [89]. Maehara et al. demonstrated that Tfh subsets expand inside and outside ectopic germinal centers in the salivary glands in IgG4-related dacryoadenitis and sialoadenitis (Mikulicz’s disease) [90]. Now, Tfh cells are divided three subsets: Tfh1 (CXCR3^+^ CCR6^−^), Tfh2 (CXCR3^−^ CCR6^−^), and Tfh17 (CXCR3^−^ CCR6^+^) cells [91]. Akiyama et al. reported that levels of Tfh2 are increased in line with serum levels of IgG4 in IgG4-RD [92]. They also reported the utility of Tfh2 as a biomarker of IgG4-RD because the number of Tfh2 cells is proportional to the risk of steroid resistance or relapse. Cargill et al. also suggested Tfh2 to be a biomarker of IgG4-related sclerosing cholangitis and type 1 AIP [93]. Among the other *T* cell subsets, CD4 and CD8 cytotoxic-T lymphocytes have been reported to play an important role in the pathophysiology of IgG4-RD [94] (Table 4).

### *B* cells

The *B* cell depletion effect of rituximab is beneficial for cases of IgG4-RD including IgG4-related pancreato-biliary disease [45–47], particularly as it reduces serum levels of IgG4 only, and not IgG1, IgG2, or IgG3 [46]. Mattoo et al. found that circulating CD19 + CD27 + CD20-CD38hi plasmablasts—which were largely IgG4 + —are increased in IgG4-RD, even in cases with normal serum levels of IgG4 [95]. The authors suggested that these plasmablasts might represent a biomarker because their number and somatic hypermutation is related to therapy response and relapse. In IgG4-cholangiopathy, levels of IgG4^+^ *B* cell receptor clones have been found to be increased in blood and tissue, although they can be eliminated with corticosteroid treatment [96].

Several surface markers have been detected on Bregs; Sumimoto et al. provided that the number of circulating CD19^+^CD24^+^CD38^high^ Bregs are increased and CD19^+^CD24^high^CD27^+^ Bregs decreased in type 1 AIP [97]. This indicates that CD19^+^CD24^high^CD38^high^ Bregs increase to inhibit disease activity, while CD19^+^CD24^high^CD27^+^ Bregs might be affected in the development of type 1 AIP. B cells therefore are considered to take a pivotal part in the pathophysiology in IgG4-RD (Table 4).

### Autoantibodies and autoantigens

The presence of autoantibodies including those to lactoferrin, carbonic anhydrase II, and pancreatic trypsin inhibitor have been reported in patients with type 1 AIP [98]. Recently, several potential autoantigens have been reported in the context of IgG4-RD including type 1 AIP: prohibitin [99], annexin A11 [100], laminin 511-E8 [101], galectin-3 [102] and anti-IL-1 receptor antagonist (IL-1RA) [103]. Prohibitin regulates mitochondrial function and is involved in the progression of several diseases including Parkinson’s and Alzheimer’s diseases, kidney diseases, cardiac diseases, and cancer [104]. Du et al. evaluated 89 patients with IgG4-RD and reported 73% to be positive for prohibitin, whereas only 1.4% of healthy control subjects were found to be positive [99]. Annexin A11 is a calcium-dependent phospholipid-binding protein, which is abundant in the nucleus [105]. Hubers et al. reported novel autoantigens of annexin A11 and IgG4-antibodies to have an anti-inflammatory role in cases of type1 AIP or IgG4-sclerosing cholangitis [100]. Shiokawa et al. reported the presence of antilaminin 511-E8 antibodies in 26 out of 51 patients with type 1 AIP and found that immunization with laminin 511-E8 could induce pancreatitis in mice [101]. Next-generation sequencing has been used to reveal the Ig gene sequence from plasmablast clones in IgG4-RD, following which, mass spectroscopy demonstrated galectin-3 to be an antigen recognized by IgG4 and IgE [102]. Galectin-3 is expressed in several cells, particularly activated macrophages and has been reported to be involved in fibrotic diseases in the liver, kidney, lung, and so on [106]. However, a large-cohort study reported the frequency of autoantigens against prohibitin, annexin A11, laminin 511-E8, and galectin-3 to be 10, 12, 7 and 28%, respectively, among patients with IgG_4_-RD [107]. Recently, the anti-IL-1RA antibody was identified as an autoantibody by sequencing plasmablast samples from IgG4-RD patients. This anti-IL-1R antibody may induce inflammation and fibrotic changes in the context of IgG4-RD as well as systemic lupus erythematosus and rheumatoid arthritis. Although their recent identification and subsequent revealing of function suggests that autoantigens have an important role in the progression and pathology of AIP, further study is necessary to make conclusive statements about their function and potential as therapeutic targets (Table 4).

### Innate immune system

Watanabe et al. provided that TLRs and nucleotide-binding oligomerization-domain-like receptors are activated in the monocytes [108] and basophils [109] in the peripheral blood in patients with IgG4-RD, and increased IgG4 production by *B* cells can occur in healthy subjects through the production of B-cell-activating factor (BAFF). Yanagawa et al. presented TLR2/TLR4-positive basophil infiltration in the pancreas of patients with type 1 AIP, and found the proportion of peripheral basophils activated by TLR4 stimulation to be significantly higher in type 1 AIP and atopic dermatitis than in healthy controls [110]. Basophils are present in target tissues where they produce Th2 cytokines in allergic disease and parasitic infection [111]. Further, Fukui et al. have demonstrated that TLR-7-positive M2-macrophages infiltrated in resected pancreata of patients with type 1 AIP abundantly [112]. Basophil has been reported to direct to the differentiation of inflammatory monocytes into M2 macrophages and to lead to the Th2 immune response in allergic disease [113]. It is thought that the same mechanisms underly the progression of type 1 AIP and IgG4-RD [110].

We have previously described neutrophil infiltration in type 1 AIP [31]. The underlying mechanism involved no significant difference in the expression of IL-8 in the pancreatic duct epithelia between types 1 and 2 AIP [31]. Arai et al. clarified the association between neutrophil extracellular traps (NETs) and IgG4 production in type 1 AIP and identified the pancreata of patients with type 1 AIP to contain NETs, which were not present in those of healthy controls. In the existence of NETs, plasmacytoid dendritic cells generated interferon-α and BAFF, and induce B cells to produce IgG4 [114]. Taken together, these results suggest that the innate immune response is participated in the development of type 1 AIP (Table 4).

### Prospects for the future of IgG4-related disease (include type 1 autoimmune pancreatitis)

**Fig. 1:**
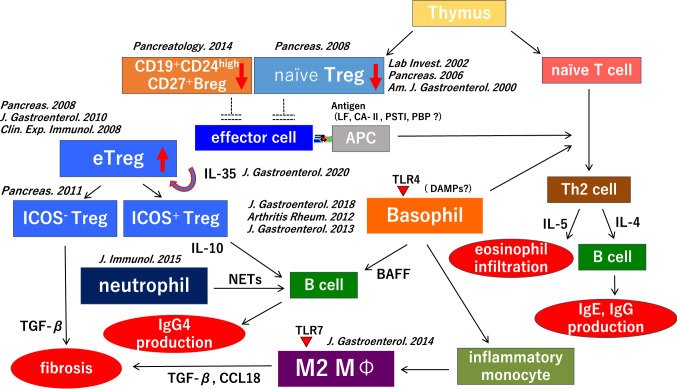
Flow chart illustrating the suggested pathophysiology of type 1 autoimmune pancreatitis (AIP). Decreased numbers of circulating naïve regulatory *T* cells and CD19^+^CD24^high^CD27^+^ regulatory *B* cells (Bregs) may participate in the initiation of type 1 AIP. Interleukin (IL)-35 stimulates the development of eTregs and progression of the disease, and an enhanced Th2 immune response. The production of immunoglobulin (Ig) G4 may be regulated through IL-10 secreted from inducible costimulator (ICOS)-positive Tregs, and basophils and monocytes also control the production of IgG4 via Toll-like receptor signaling. M2 macrophages and tumor growth factor-β secreted from ICOS-negative Tregs may accelerate fibrosis. M2 macrophages may also involve Th2 immune response in type 1 AIP. Neutrophils also affect IgG4 production through neutrophil extracellular traps (NETs)

From our own research, we suggest the following pathophysiology of type 1 AIP: first, naïve regulatory *T* cells and CD19^+^CD24^high^CD27^+^ regulatory *B* cells play an important role in the initial stages of disease. Following a decrease in these cells, IL-35 stimulates the development of eTregs, which can be divided into ICOS-positive and ICOS-negative Tregs. Regulatory *T* cells produce IL-10 and TGF-ß, which induce *B* cells to transform to IgG4-producing plasma cells and fibrosis, respectively. In innate immunity, basophils cause inflammatory monocytes to differentiate into M2 macrophages, affecting the production of IgG4 via TLR signaling and influencing the Th2 immune environment. Also, M2 macrophages contribute to the development of fibrosis and stimulation of the Th2 immune reaction. Neutrophils also influence IgG4 production via NETs (Fig. 1).

However, many clinical and fundamental issues remain unclear including the diagnosis, treatment, recurrence, prognosis, and pathogenesis. Moreover, in the seronegative and focal types, it seems to be challenging completely to remove cases in which it is impossible to distinguish between pancreatic cancer and type 1 AIP using the present diagnostic tools. Relapses are also a serious problem in the clinical practice, because patients who do not require maintenance therapy can avoid unnecessary treatment. There is an urgent need to develop a specific biomarker for the diagnosis and prediction of relapse, including a liquid biopsy.

## Conclusion

Type 1 AIP is recognized as pancreatic lesions in IgG4-RD. We believe that this article provides a foundation for further investigation to clarify a number of these issues.
